# Transcriptomic and Metabolomic Analyses Provide Insights into the Formation of the Peach-like Aroma of *Fragaria nilgerrensis* Schlecht. Fruits

**DOI:** 10.3390/genes13071285

**Published:** 2022-07-20

**Authors:** Ai-Hua Wang, Hong-Ye Ma, Bao-Hui Zhang, Chuan-Yuan Mo, En-Hong Li, Fei Li

**Affiliations:** 1 Horticulture Institute (Guizhou Horticultural Engineering Technology Research Center), Guizhou Academy of Agricultural Sciences, Guiyang 550006, China; 118wah@163.com (A.-H.W.); lyhmyyqh@163.com (H.-Y.M.); 18786102250@163.com (B.-H.Z.); mochuanyuan@163.com (C.-Y.M.); 2 College of Biological and Food Engineering, Suzhou University, Suzhou 234099, China; 3 Guizhou Seed Management Station, Guiyang 550001, China; lienhong1982@163.com

**Keywords:** *Fragaria nilgerrensis* Schlecht., transcriptome, peach aroma, fatty acid metabolism, δ-octalactone, lactone biosynthesis

## Abstract

*Fragaria nilgerrensis* Schlecht. is a wild diploid strawberry species. The intense peach-like aroma of its fruits makes *F. nilgerrensis* an excellent resource for strawberry breeding programs aimed at enhancing flavors. However, the formation of the peach-like aroma of strawberry fruits has not been comprehensively characterized. In this study, fruit metabolome and transcriptome datasets for *F. nilgerrensis* (HA; peach-like aroma) and its interspecific hybrids PA (peach-like aroma) and NA (no peach-like aroma; control) were compared. In total, 150 differentially accumulated metabolites were detected. The K-means analysis revealed that esters/lactones, including acetic acid, octyl ester, δ-octalactone, and δ-decalactone, were more abundant in HA and PA than in NA. These metabolites may be important for the formation of the peach-like aroma of *F. nilgerrensis* fruits. The significantly enriched gene ontology terms assigned to the differentially expressed genes (DEGs) were fatty acid metabolic process and fatty acid biosynthetic process. Twenty-seven DEGs were predicted to be associated with ester and lactone biosynthesis, including *AAT*, *LOX*, *AOS*, *FAD*, *AIM1*, *EH*, *FAH*, *ADH*, and cytochrome P450 subfamily genes. Thirty-five transcription factor genes were predicted to be associated with aroma formation, including *bHLH*, *MYB*, *bZIP*, *NAC*, *AP2*, *GATA*, and *TCP* *family members*. Moreover, we identified differentially expressed *FAD*, *AOS*, and cytochrome P450 family genes and *NAC*, *MYB*, and *AP2* transcription factor genes that were correlated with δ-octalactone and δ-decalactone. These findings provide key insights into the formation of the peach-like aroma of *F. nilgerrensis* fruits, with implications for the increased use of wild strawberry resources.

## 1. Introduction

Strawberry (*Fragaria* sp.) is cultivated worldwide. Aroma, which is one of the major quality-related characteristics of strawberry, is the most important quality indicator for consumers [1]. Modern cultivated strawberry varieties are mostly derived from *Fragaria* × *ananassa*, which produces high yields of large, red, and firm fruits, but there is relatively little diversity in their aromas [2,3]. Wild species have accumulated a broader range of volatile compounds [2]. Because of the substantial diversity and strength of the aromas of natural and domesticated populations, plant breeders consider wild strawberries to be important donors of novel aromatic compounds [4]. *Fragaria nilgerrensis* Schlecht. is a wild diploid strawberry species endemic to the eastern and southeastern regions of Asia; it is an excellent material for breeding strawberry varieties with enhanced flavors because of its intense peach-like aroma [5].

Most of the studies on the fruity aromas of strawberries still focus on identifying volatile compounds and determining the differences in these compounds among strawberry cultivars [6]. The volatile compounds detected in strawberry include esters, aldehydes, ketones, alcohols, terpenes, furanones, and sulfur compounds [7]. Esters are the largest group of aromatic compounds in strawberry, and their contents and compositions vary considerably among strawberry species and cultivars [1]. Usually, different species have their own characteristic esters [8]. Methyl anthranilate is a major aroma compound in *Fragaria vesca* L., whereas methylbutanoate, ethyl butanoate, ethyl hexanoate, and methyl 2-methylbutanoate are among the most important odorants in *F. × ananassa* [9,10]. The aroma compound composition differs between *F. nilgerrensis* and *F. × ananassa*, with methyl benzoate, benzyl acetate, methyl cinnamate, and ethyl cinnamate identified as the main compounds in the former [11]. Previous studies revealed that *F. nilgerrensis* might be an important donor of aromatic compounds, including the lactones that provide strawberry fruits with a peach-like aroma [12,13]. Because aroma is a quantitative trait, the peach-like scent of *F. nilgerrensis* fruits may be the result of the interactions between multiple metabolites. Thus, further characterizing the development of the peach-like aroma of *F. nilgerrensis* fruits is necessary for the breeding of strawberry varieties that produce fruits with desirable aromas, but there has been limited related research.

Because of the importance of volatile compounds in fruits, the mechanisms regulating volatile biosynthesis must be clarified. Transcriptional regulation is the most important form of regulation in plants. Integrating transcriptome data with metabolic profiles is a useful strategy for analyzing the synthesis of aromatic compounds as well as the associated genes [14]; however, there has been minimal related research on strawberry. It is generally believed that fruit aromatic formation involves various pathways, including those related to fatty acids, amino acids, terpenoids, and carotenoids [15]. Accordingly, many studies have focused on identifying and functionally annotating the key genes in these pathways to elucidate the mechanism underlying the formation of specific aromatic compounds. To date, only a few genes related to aroma compound synthesis have been identified in strawberry. For example, *FaFAD1* encodes a fatty acid desaturase that controls γ-decalactone production [12,16]. Other studies indicated *FanAAMT* is involved in the production of methyl anthranilate [17], whereas *FaOMT* contributes to mesifurane synthesis [18] and *FaNES1* mediates linalool synthesis [19]. Additionally, some genes encoding transcription factors (TFs) that help to regulate aroma formation pathways have been identified in strawberry [20]. Thus, the regulated formation of aromatic compounds in strawberry fruits is a complex process. These earlier studies increased our understanding of the links between genes and metabolites, but they were insufficient for deciphering the regulatory mechanism underlying strawberry aroma formation. Many key genes, including those involved in known aroma compound synthesis pathways, still need to be identified and functionally characterized.

In this study, *F. nilgerrensis* (HA) fruits, which have a peach-like aroma, and fruits from the interspecific hybrids PA (with a peach-like aroma) and NA (without a peach-like aroma) were examined. Their volatile compounds were detected by headspace solid-phase microextraction coupled with gas chromatography/mass spectrometry (HS-SPME-GC/MS). Additionally, their transcriptomes were analyzed by RNA-seq. The results of this study provide insights into the formation of the peach-like aroma of *F. nilgerrensis* fruits, which may be useful for maximizing the utility of wild strawberry resources.

## 2. Materials and Methods

### 2.1. Fruit Materials

Ripe fruit samples were collected from *F. nilgerrensis* Schlecht. (with a peach-like aroma) and its interspecific hybrids PA (with a peach-like aroma) and NA (without a peach-like aroma) at the Germplasm Strawberry Repository of the Guizhou Horticulture Institute located in Guiyang (26.492310° N, 106.653870° E), Guizhou province, China. Six biological replicates were collected in 2021. The fruit samples were immediately frozen in liquid nitrogen and then stored at −80 °C.

The interspecific hybrids PA and NA were generated as follows:
Benihoppe (female, 8*x*) × *F. nilgerrensis* Schlecht. (male, 2*x*)       ↓      5*x*
↓ Chromosome doubling     BF (female, 10*x*) ↓ Seedling selection      PA and NA

### 2.2. Qualitative and Quantitative Analyses of Extracted Volatiles

Frozen fruit samples were ground to a powder in liquid nitrogen and then 1 g powder was transferred to a 20 mL headspace vial (Agilent, Palo Alto, CA, USA) containing a saturated NaCl solution and 10 μL Furfural-3,4,5-d3 (CAS-53599-40-9), which was used as an internal standard. The vials were sealed using crimp-top caps with TFE-silicone headspace septa (Agilent). For the SPME analysis, each vial was incubated at 100 °C for 5 min and then 120 µm divinylbenzene/carboxen/polydimethylsiloxane fiber (Agilent) was inserted into the headspace for 15 min at 100 °C.

Volatile organic compounds were identified and quantified using the 8890 gas chromatograph and the 5977B mass spectrometer (Agilent) equipped with a 30 m × 0.25 mm × 0.25 μm DB-5MS (5% phenyl-polymethylsiloxane) capillary column. Helium was used as the carrier gas at a linear velocity of 1.0 mL/min. The injector and detector temperatures were set at 250 and 280 °C, respectively. The oven temperature program was as follows: 40 °C for 3.5 min, increase to 100 °C at 10 °C/min, increase to 180 °C at 7 °C/min, increase to 280 °C at 25 °C/min, and then hold for 5 min. Mass spectra were recorded in the electron impact ionization mode at 70 eV. The quadrupole mass detector, ion source, and transfer line temperatures were set at 150, 230, and 280 °C, respectively. Mass spectra were scanned in the range m/z 50–500 amu at 1 s intervals. The volatile compounds corresponding to the mass spectra were identified using data system libraries (MWGC or NIST) and the linear retention index. Differentially accumulated metabolites (DAMs) were identified for each comparison (PA vs. NA and HA vs. NA) using the following thresholds: Variable Importance in the Projection (VIP) value >1.0, fold-change >2.0 or <0.5, and *p* < 0.05.

### 2.3. Total RNA Extraction and Sequencing

Total RNA was extracted from 18 fruit samples using the TRIzol Plant RNA Extraction Kit (Thermo Fisher Scientific, Waltham, MA, USA). A NanoDrop 2000 spectrophotometer (Thermo Scientific, Pittsburgh, PA, USA) was used to determine the purity, concentration, and integrity of the RNA samples. High-quality RNA was used to construct cDNA libraries, which were subsequently sequenced using the Illumina HiSeq platform to generate 125-bp/150-bp paired-end reads.

### 2.4. Transcriptome Analysis

The raw data were filtered using fastp (version 0.19.3) to eliminate low-quality reads. The clean reads were mapped to the downloaded *F. vesca* L. genome and its annotation files (https://www.ncbi.nlm.nih.gov/genome/?term=Fragaria+vesca+L (accessed on 3 July 2021)) by HISAT v2.1.0. Use StringTie v1.3.4d for new gene prediction. Additionally, gene expression levels were calculated in terms of fragments per kilobase per million (FPKM) values by featureCounts v1.6.2. The DESeq2 program (version 1.22.1) was used to analyze genes that were differentially expressed between two sample groups (PA vs. NA and HA vs. NA), with the *p*-value corrected according to the Benjamini–Hochberg method [21]. The corrected *p*-value and fold-change were used to detect significant differences in expression. The differentially expressed genes (DEGs) in each comparison (PA vs. NA and HA vs. NA) were identified on the basis of the following criteria:|fold change| ≥ 1.2 and false discovery rate (FDR) < 0.05. The hypergeometric distribution test was performed to identify the enriched Kyoto Encyclopedia of Genes and Genomes (KEGG) pathways (https://www.kegg.jp/ (accessed on 23 June 2021)) and Gene Ontology (GO) terms (http://www.geneontology.org/ (accessed on 24 May 2021)) among the DEGs.

### 2.5. Quantitative Real-Time Polymerase Chain Reaction (qRT-PCR) Analysis

To verify the accuracy and reliability of the transcriptome sequencing data, 8 DEGs were randomly selected for a qRT-PCR assay. First-strand cDNA was synthesized using the PrimeScript™ RT reagent Kit (TaKaRa, Dalian, China). The qRT-PCR assay was completed using TB Green Premix Ex Taq™ II (TaKaRa) and the BioRad CFX96 Real-Time PCR System (Bio-Rad Laboratories, Inc., Hercules, CA, USA). The qRT-PCR primer sequences designed by Primer 6.0 are listed in Table S1. The relative expression levels of the DEGs were calculated using the comparative Ct (2^−ΔΔCt^) method after normalizing the expression data to the *FaActin* internal transcript control [22].

### 2.6. Correlation Analysis of the Transcriptome and Metabolome Data

For the combined analysis of the metabolome and transcriptome data, the COR program from R was used to calculate Pearson’s correlation coefficient (PCC). The corresponding correlation network was visualized using the Cytoscape software (version 3.7.0). Strong correlations were determined on the basis of the following: PCC ≥ 0.8 and *p* ≤ 0.05.

## 3. Results

### 3.1. Metabolomic Profiling

**Figure 1:**
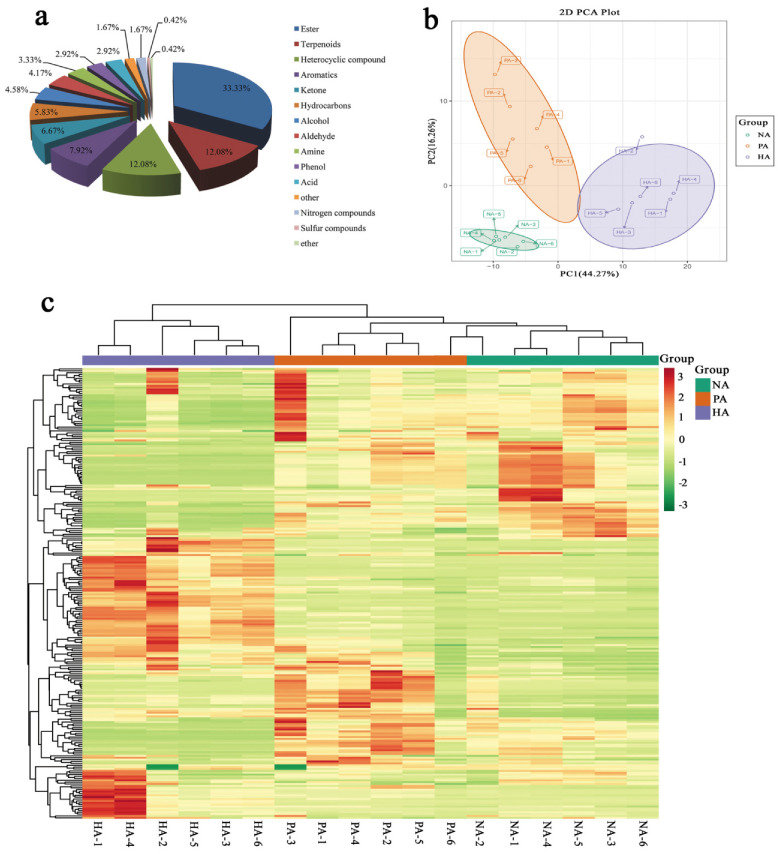
Analysis of all metabolites. Metabolite types (**a**). Two-dimensional principal component analysis plot (**b**). Heatmap for the hierarchical clustering analysis (**c**).

The HS-SPME-GC/MS analysis resulted in the identification of 240 volatile compounds in the 18 analyzed samples. These compounds included 80 esters, 29 terpenoids, 29 heterocyclic compounds, 19 aromatics, 16 ketones, 14 hydrocarbons, 11 alcohols, 10 aldehydes, eight amines, seven phenols, seven acids, four nitrogen compounds, one sulfur compound, one ether, and four other compounds. Thus, esters were the most abundant compounds (33.33%), followed by terpenoids (12.08%), heterocyclic compounds (12.08%), and aromatics (7.92%) (Figure 1a). A principal component analysis revealed the overall differences in the metabolites among the sample groups as well as the variability within sample groups. Principal components 1 and 2 respectively explained 44.27% and 16.26% (i.e., 60.53% combined) of the total variance among samples. The metabolites of the NA samples differed significantly from those of the PA and HA samples. Additionally, there was some overlap between the metabolites of the PA and HA samples, possibly because both PA and HA fruits have a peach-like aroma (Figure 1b). The heatmap hierarchical clustering results revealed that the biological replicates were grouped together and the metabolite contents varied substantially among NA, PA, and HA (Figure 1c). These findings indicated that the analysis was appropriate and the metabolome data were highly reliable.

### 3.2. Identification of DAMs

**Figure 2:**
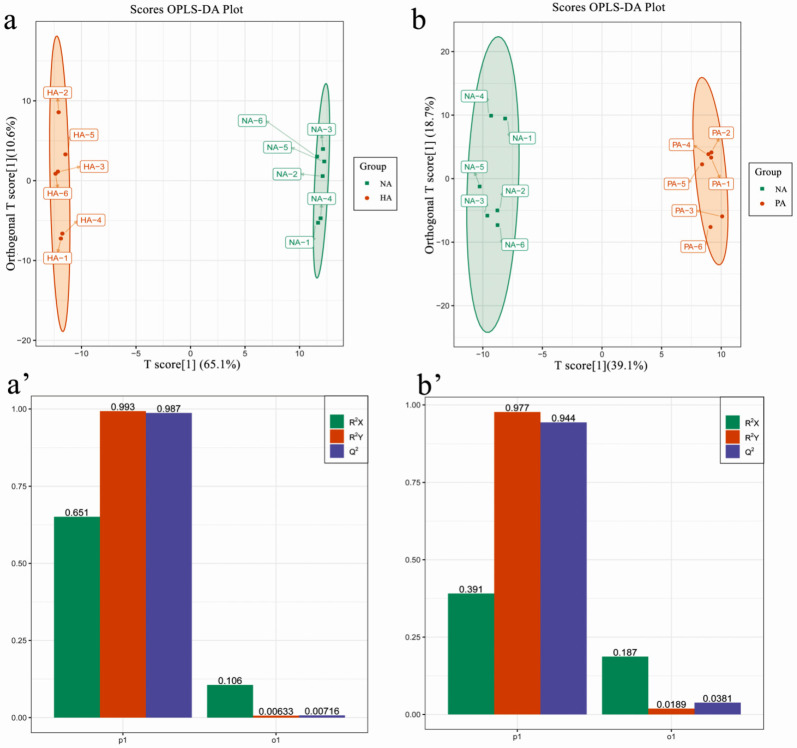
Metabolomics profiling. Orthogonal partial least squares-discriminant analysis (OPLS-DA) of NA vs. HA (**a**) and NA vs. PA (**b**). The 200-response sorting tests of the OPLS-DA model for NA vs. HA (**a′**) and NA vs. PA (**b′**). Q^2^ is an important parameter for evaluating the OPLS-DA model. R^2^X and R^2^Y represent the percentage of the OPLS-DA model that can explain the X and Y matrix information, respectively.

**Figure 3:**
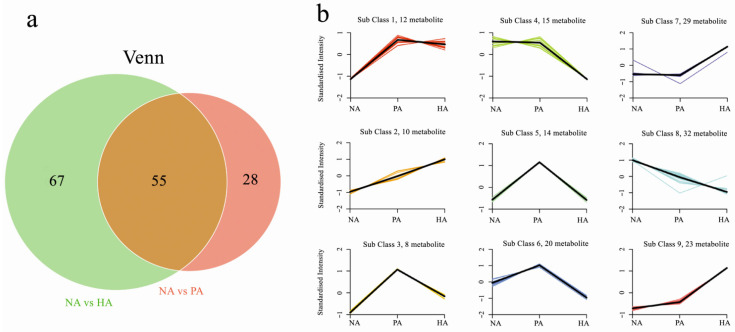
Venn diagram (**a**) and K-means (**b**) analyses of differentially abundant metabolites.

To identify DAMs among the examined samples, VIP ≥1 and fold-change ≥2 or ≤0.5 were used as the thresholds. The results of the orthogonal partial least squares-discriminant analysis (OPLS-DA) (Figure 2a,b) and the 200-response sorting tests (Figure 2a′,b′) indicated that the model was stable and reliable. Moreover, the VIP analysis could be used to screen for DAMs. A total of 150 DAMs were revealed by the NA vs. HA and NA vs. PA comparisons (Table S2). There were 55 DAMs that were common to both comparisons, whereas 67 and 28 DAMs were exclusive to the NA vs. HA and NA vs. PA comparisons, respectively. Thus, more DAMs were detected in the NA vs. HA comparison than in the NA vs. PA comparison (Figure 3a). To study the change trend of relative contents of metabolites in different samples, the relative contents of different metabolites were standardized and centralized and then analyzed by K-means clustering. The results showed that the K-means analysis divided the 150 DAMs into nine clusters (Figure 3b). The metabolites of Clusters 1, 2, 3, and 9 were significantly more abundant in PA and HA than in NA. These four clusters contained 53 metabolites, of which 29 were esters/lactones, including acetic acid, octyl ester, hexanoic acid, 1-methylethyl ester, octanoic acid, methyl ester, octanoic acid, octyl ester, δ-octalactone, and δ-decalactone (Table S3).

### 3.3. RNA-seq Analysis and Assembly and Functional Annotation

Three biological replicates of the HA, PA, and NA fruit samples were used for an Illumina RNA-seq analysis. Nine cDNA libraries were constructed and sequenced. After the raw reads were filtered, 400.45 million high-quality clean reads (60.09 billion bases) remained. More specifically, the Q20 and Q30 values for each library exceeded 97.49% and 92.99%, respectively. The GC content ranged from 45.45 to 46.54% (Table 1).

To verify the RNA-seq results, the expression levels of 8 randomly selected DEGs were analyzed in a qRT-PCR assay, which indicated that the changes in the expression of these 8 genes were basically consistent with the RNA-seq data (Figure S1). Hence, the RNA-seq data were accurate and reliable.

### 3.4. Identification and Functional Analysis of DEGs

**Figure 4:**
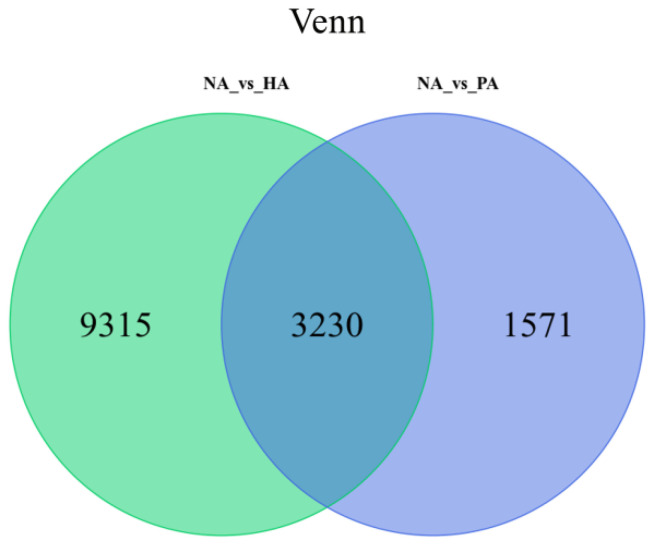
Venn diagram analysis of differentially expressed genes.

The RNA-seq data were compared as follows to identify DEGs among the examined fruit samples: NA vs. HA and NA vs. PA. A total of 14,116 DEGs were identified, of which 3230 were common to both comparisons, whereas 9315 and 1571 were specific to the NA vs. HA and NA vs. PA comparisons, respectively. A Venn diagram was used to illustrate the number of DEGs in both comparisons (Figure 4). These results suggested that the DEGs revealed by the two comparisons may be related to the peach-like aroma of HA and PA fruits.

**Figure 5:**
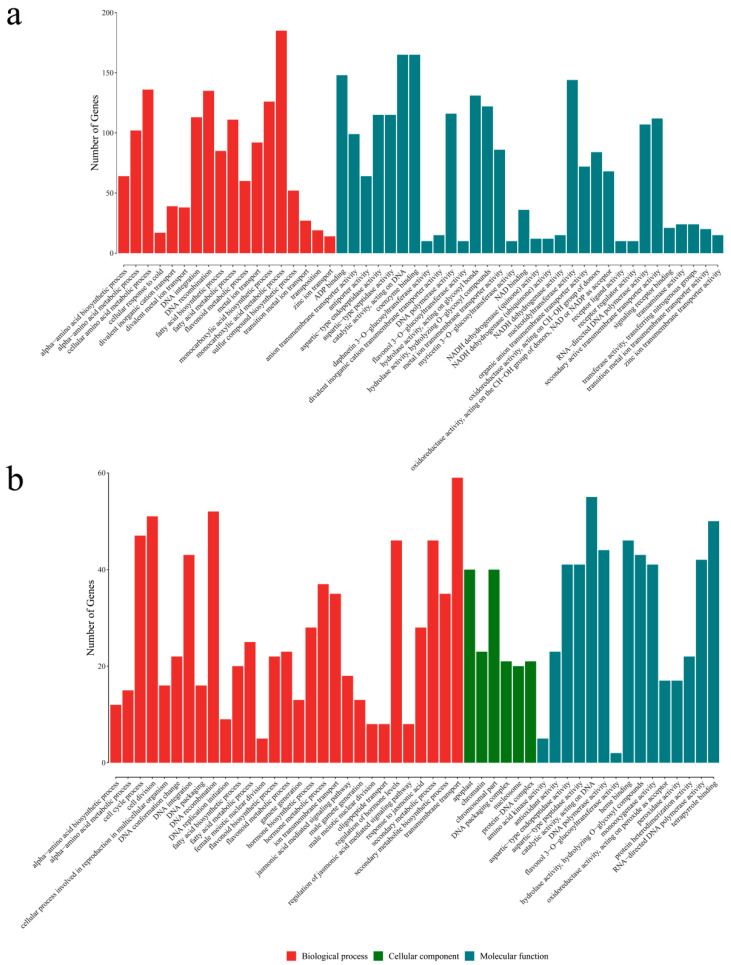
GO analysis of differentially expressed genes (**a**,**b**).

To clarify the biological functions of the DEGs, the significant DEGs identified by the two comparisons were functionally categorized according to GO and KEGG enrichment analyses. The GO functional analysis indicated that the DEGs in the NA vs. HA comparison was associated with diverse biological processes (18 sub-categories) and molecular functions (32 sub-categories) (Figure 5a), whereas the DEGs in the NA vs. PA comparison were related to biological processes (25 sub-categories), cellular components (six sub-categories), and molecular functions (19 sub-categories) (Figure 5b). Several aroma component synthesis-related GO terms were assigned to the DEGs in both comparisons, including flavonoid metabolic process, fatty acid metabolic process, fatty acid biosynthetic process, α-amino acid biosynthetic process, α-amino acid metabolic process, and flavonol 3-O-glucosyltransferase activity.

**Figure 6:**
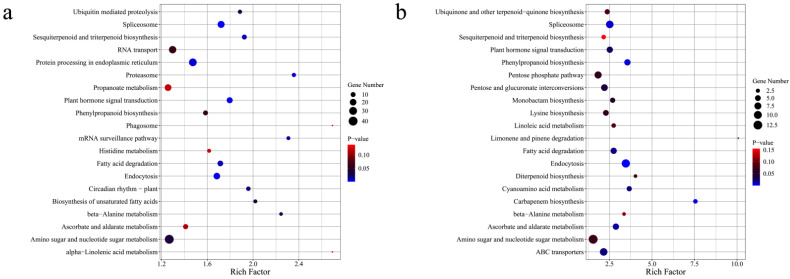
KEGG analysis of differentially expressed genes (**a**,**b**).

The 20 most enriched KEGG pathways among the DEGs revealed by the NA vs. HA and NA vs. PA comparisons were determined (Figure 6a,b). The following six pathways were enriched among the DEGs in both comparisons: sesquiterpenoid and triterpenoid biosynthesis, phenylpropanoid biosynthesis, fatty acid degradation, β-alanine metabolism, ascorbate, and aldarate metabolism, and amino sugar and nucleotide sugar metabolism. Particularly, fatty acids are major precursors of aroma volatiles in most fruit [15], the DEGs related to fatty acid degradation in the two comparisons, α-linolenic acid metabolism in NA vs. HA comparison (Figure 6a), and linoleic acid metabolism in NA vs. PA comparison (Figure 6b) may be associated with ester/lactone synthesis in strawberry.

### 3.5. Expression Patterns of Structural DEGs Related to Ester and Lactone Biosynthesis

**Figure 7:**
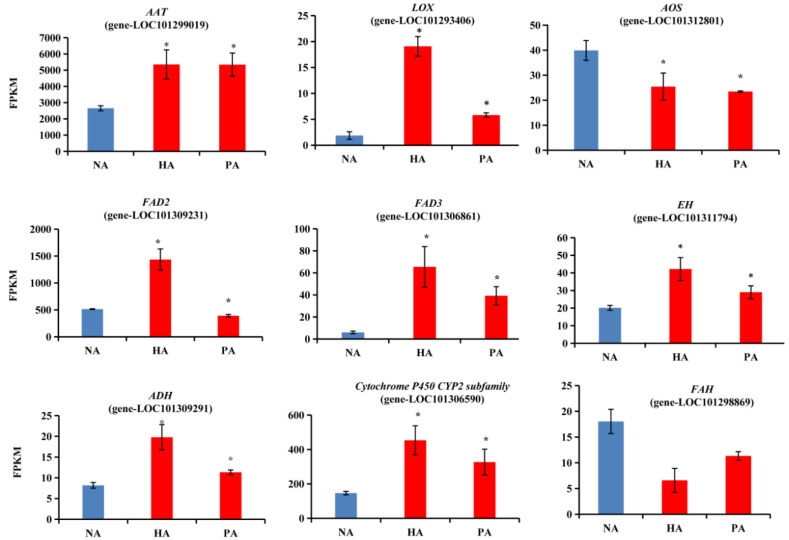
Relative expression levels of the structural DEGs related to ester and lactone biosynthesis. Significant differences in expression levels are indicated by an asterisk.

The 27 DEGs predicted to be associated with the biosynthesis of esters and lactones comprised two alcohol acyltransferase (*AAT*) genes, two lipoxygenase (*LOX*) genes, one allene oxide synthase (*AOS*) gene, three fatty acid desaturase (*FAD*) genes, one hydroxyacyl-CoA dehydrogenase (*AIM1*) gene, two epoxide hydrolase (*EH*) genes, one fatty acid hydroxylase (*FAH*) gene, 11 cytochrome P450 subfamily genes, and four alcohol dehydrogenase (*ADH*) genes (Table S4, Figure 7). The *ATT*, *LOX*, *EH*, cytochrome P450 subfamily and *ADH* genes had up-regulated expression levels, which was in contrast to the down-regulated expression levels of the *AOS* and *FAH* genes. Interestingly, the omega-6 fatty acid desaturase (*FAD2*) and omega-3 fatty acid desaturase (*FAD3*) gene expression trends differed (Figure 7). Therefore, these candidate genes will need to be further analyzed to elucidate their potential roles in the molecular mechanisms mediating the formation of the peach-like aroma of *F. nilgerrensis* fruits.

### 3.6. Expression Patterns of TF Genes Involved in Regulating Aroma Formation

**Figure 8:**
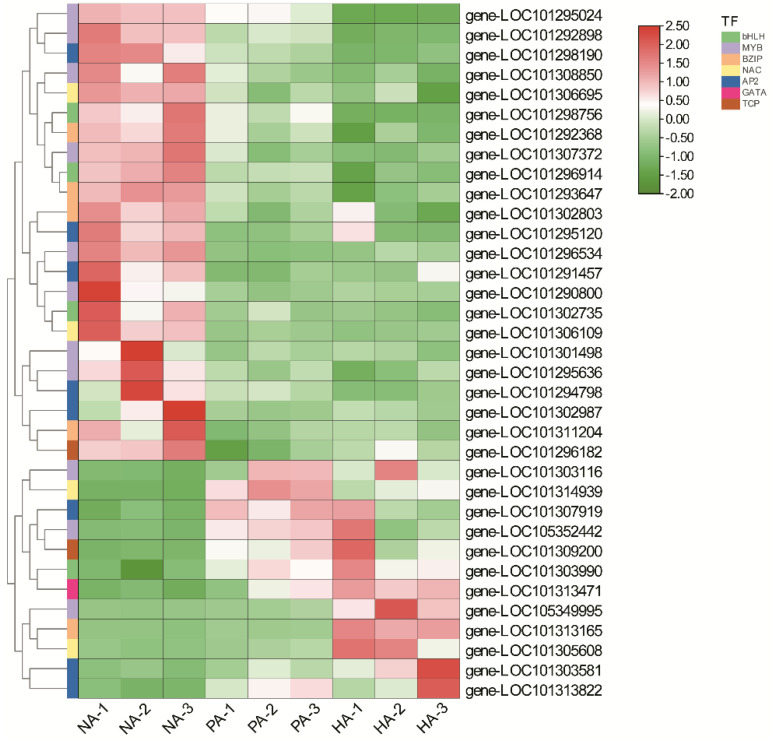
Expression patterns of genes encoding TFs are involved in the regulation of aroma formation.

The significant DEGs included 35 genes encoding TFs that were predicted to be associated with aroma formation. Of these DEGs, 12 and 23 had up-regulated and down-regulated expression levels, respectively (Table S5). Additionally, these genes encoded the following: four bHLH TFs, 11 MYB TFs, five bZIP TFs, four NAC TFs, eight AP2 TFs, one GATA TF, and two TCP TFs (Figure 8). Among the TF genes, *TCP9* (LOC101309200) expression was up-regulated 36.85- and 31.62-fold, *MYB* (LOC105349995) expression was up-regulated 34.02- and 3.95-fold, *AP2* (LOC101302987) expression was down-regulated 4.89- and 19.15-fold and *bZIP* (LOC101311204) expression was down-regulated 3.78- and 7.72-fold in the NA vs. HA and NA vs. PA comparisons, respectively (Figure 8), implying they may play a critical role in the formation of the peach-like aroma of *F. nilgerrensis* fruits.

### 3.7. Correlation between the Transcriptome and Metabolome Data

**Figure 9:**
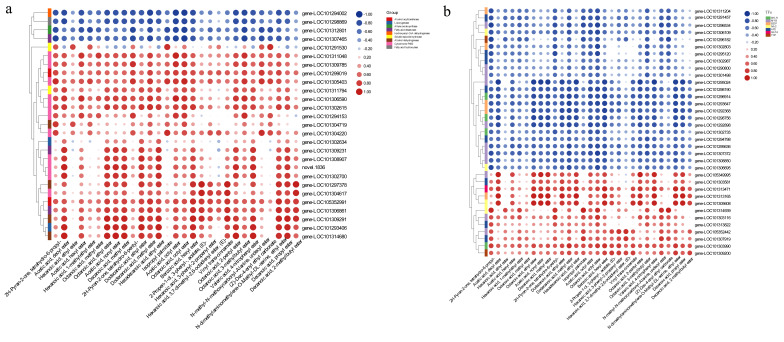
Analyses of the correlation between 27 key structural genes and 29 key metabolites (**a**) and between 35 key TFs and 29 key metabolites (**b**).

To further clarify the relationships between the key DEGs and DAMs, we analyzed whether the 29 esters/lactones were more abundant in the HA and PA fruits (i.e., with a peach-like aroma) than in the NA fruits (i.e., without a peach-like aroma) were correlated with 27 structural DEGs related to ester/lactone biosynthesis or 35 TF-encoding DEGs. Among the structural genes, *AIM1*, *FAH*, and *AOS* were negatively correlated with the esters/lactones, whereas *AAT*, cytochrome P450 family genes, and *ADH* were positively correlated with esters/lactones (Figure 9a, Table S6). Of the TF genes, only the *GATA* family genes were positively correlated with esters/lactones. In contrast, the *bHLH*, *MYB*, *bZIP*, *NAC*, *AP2*, and *TCP* family members were differentially correlated with esters/lactones (Figure 9b, Table S7). We subsequently focused on the structural genes and TF genes associated with two lactones.

**Figure 10:**
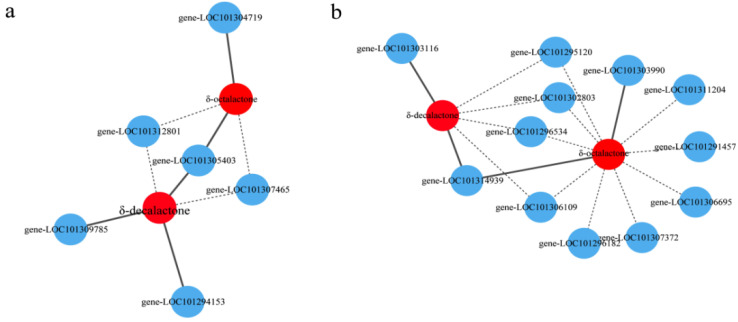
Analyses of the correlation between key structural genes and two key lactones (**a**) and between TFs and two key lactones (**b**). Blue circles represent the differentially expressed genes and TFs, whereas red circles represent the two key lactones. The solid and dotted lines indicate positive and negative correlations, respectively.

The four structural genes significantly correlated with δ-octalactone were *FAD* (LOC101307465), *AOS* (LOC101312801), *ADH* (LOC101304719), and a cytochrome P450 family member (LOC101305403); the correlation was positive for *ADH* (Figure 10a, Table S6). The five structural genes significantly correlated with δ-decalactone were *FAD* (LOC101307465), *AOS* (LOC101312801), and three cytochrome P450 family members (LOC101294153, LOC101305403, and LOC101309785); the correlation was positive for the three cytochrome P450 family members (Figure 10a, Table S6). Eleven TF genes were strongly correlated with δ-octalactone, including genes in the *NAC* (LOC101306695, LOC101306109 and LOC101314939), *MYB* (LOC101296534 and LOC101307372), *bZIP* (LOC101302803 and LOC101311204), *AP2* (LOC101295120 and LOC101291457), *TCP* (LOC101296182), and *bHLH* (LOC101303990) families; the correlation was positive for *NAC* (LOC101314939) and *bHLH* (LOC101303990) (Figure 10b, Table S7). Six TF genes were strongly correlated with δ-decalactone, including genes belonging to the *NAC* (LOC101306109 and LOC101314939), *MYB* (LOC101296534 and LOC101303116), bZIP (LOC101302803), and *AP2* (LOC101295120) families; the correlation was positive for *NAC* (LOC101314939) and *MYB* (LOC101303116) (Figure 10b, Table S7). Accordingly, the TFs encoded by these genes might regulate the synthesis of aromatic compounds by controlling the expression of key genes involved in aroma compound synthesis pathways.

## 4. Discussion

### 4.1. Metabolome Analysis

*F. nilgerrensis* is a suitable donor for improving the flavor-related qualities of cultivated strawberry varieties through breeding [23]. For example, Noguchi used *F. nilgerrensis* and *F. × ananassa* to obtain an interspecific decaploid hybrid (‘Tokun’) that produces fruits with a unique blend of peach-like and coconut-like aromas [24]. However, little is known about the composition of the aromatic compounds that provide *F. nilgerrensis* fruits with their characteristic peach-like aroma. In the present study, we analyzed the aroma-related metabolic profiles of the ripe fruits from *F. nilgerrensis* (HA) and its interspecific hybrids PA (with a peach-like aroma) and NA (without a peach-like aroma). The results showed that 29 esters were more abundant in PA and HA than in NA (Table S3). Esters are a very important component of strawberry aromas [1,25]. Consistent with previous studies, this study also detected two esters unique to *F. nilgerensis* fruits, namely octanoate, ethyl ester, and trans-4-decanoate ethyl ester [26]. Lactones also influence the aromatic characteristics of *F. nilgerrensis* fruits and provide fruits with a fruity, sweet, or peach-like aroma and flavor [13,27]. Zhao et al. (2014) identified δ-decalactone and γ-dodecalactone as two lactones responsible for the characteristic aroma of *F. nilgerrensis* fruits, with γ-dodecalactone contributing substantially to the peach-like scent [26]. Additionally, δ-decalactone was detected as one of the compounds associated with the characteristic aroma of peach fruits [28]. Consistent with the findings of these earlier studies, the δ-decalactone content was revealed to be significantly higher in PA and HA fruits than in NA fruits, implying it may be crucial for the formation of the peach-like aroma of *F. nilgerrensis* fruits. Previous research confirmed γ-decalactone is another lactone related to the peach-like flavor of strawberry fruits [12,29] and also one of the compounds that contributed most to the aroma of peach juice [30]. In contrast with earlier studies, γ-decalactone and γ-dodecalactone were not significantly or down-regulated in *F. nilgerrensis* (Table S2), respectively. Therefore, whether they are the primary aromatic compounds responsible for the characteristic aroma of *F. nilgerrensis* fruits remains to be confirmed. Moreover, In this study, the NA vs. HA and NA vs. PA comparisons revealed δ-octalactone was among the lactones that were more abundant in HA and PA than in NA. We believe that it may also be related to the peach-like aroma of *F. nilgerrensis* fruits. Therefore, the significant increase in δ-decalactone and δ-octalactone contents may be critical for the formation of the peach-like aroma of *F. nilgerrensis* fruits. The characteristic aroma of strawberry fruits may be related to the changes in the proportion of key aroma components [31]. Furthermore, other esters, such as acetic acid, octyl ester, may also contribute to the formation of the peach-like aroma to some extent [6].

### 4.2. Transcriptome Analysis

Strawberry has highly complex fruit aromas, which are the result of hundreds of volatile compounds [32]. However, only a few genes and TFs controlling the formation of these volatile compounds have been identified [19,33,34,35], and genes responsible for the peach-like aroma of *F. nilgerrensis* fruits have hardly been reported. In the present study, 27 DEGs were associated with ester and lactone biosynthesis (Table S4). The KEGG and GO analyses indicated most of these genes (e.g., *LOX*, *ADH*, *AAT*, *FAH*, and *FAD*) are involved in fatty acid pathways (Figure 5 and Figure 6). Both omega-6 fatty acid desaturase and omega-3 fatty acid desaturase may be associated with the production of a precursor of lactones/esters [36,37]. Interestingly, the genes encoding these two enzymes had the opposite expression patterns, suggesting they may have different roles in the formation of strawberry fruit aromas. This phenomenon is similar to that observed in previous studies examining peach fruit volatile formation [12,37]. According to published reports, the accumulation of γ-decalactone, which has a peach-like flavor, is highly correlated with the activities of AAT, FAD, FAH, ACX, and cytochrome P450 hydroxylases [12,16,27,38,39,40]. Additionally, the observed increase in the lactone content following the transient over-expression of *Mi9LOX* and *MiEH2* reflected their probable role in the lactone biosynthesis in mango fruits [41]. Therefore, the above-mentioned genes may be important for the formation of the peach-like aroma of *F. nilgerrensis* fruits. Previous studies revealed that several TFs, such as NAC, ERF, bHLH, MYB, bZIP, TCP, GATA, and HSF family members, help regulate the biosynthesis of fruit flavor-related components [42,43,44,45,46]. Recent research demonstrated that AP2 and FaMYB9 are key TFs that regulate the production of aromatic compounds in strawberry [20,35]. In this study, 35 TFs that reportedly mediate the biosynthesis of compounds associated with fruit flavors were identified (Table S5). These results may help elucidate the regulatory factors involved in the formation of the peach-like aroma of *F. nilgerrensis* fruits.

### 4.3. Key Metabolites and Candidate Genes Associated with the Peach-like Aroma

Metabolites are the intermediate or final products of the cell biological regulatory process [47], and their accumulation, which substantially influences fruit flavor-related traits, is controlled by many functional genes and TFs. Transcriptomics and metabolomics experimental techniques have been applied to elucidate the metabolic pathways of interest in many plants [48,49,50,51]. To clarify the formation of the peach-like aroma of *F. nilgerrensis* fruits, we performed an integrated analysis of the transcriptome and metabolome, which identified many DEGs involved in linoleic acid metabolism, α-linolenic acid metabolism, and fatty acid degradation as well as the production of many DAMs of esters and lactones. Two lactones related to the peach-like aroma (δ-decalactone and δ-octalactone) were identified in this study, but the genes encoding these two lactones and the related factors that regulate the expression of these genes have not been reported. A total of 18 DEGs were significantly correlated with these two lactones in strawberry, including functional genes (*AOS*, *ADH*, *FAD*, and cytochrome P450 family members) and TF-encoding genes (*MYB*, *NAC*, *TCP*, *bZIP*, *bHLH*, and *AP2*) (Figure 10). These findings may reflect the complexity of the regulatory mechanism linking the changes in metabolite accumulation and gene expression in strawberry. To improve our understanding of the mechanism mediating the formation of the peach-like aroma of *F. nilgerrensis* fruits, the candidate gene functions, and the related regulatory mechanisms will need to be characterized via gene over-expression or silencing experiments as well as ChIP-seq and yeast one- or two-hybrid assays. Notably, we revealed that two *NAC* family genes (LOC101302803 and LOC101314939) are significantly correlated with two lactones in strawberry fruits for the first time. This suggests that the *NAC* gene family might be critical for the formation of the peach-like aroma of *F. nilgerrensis* fruits. Considered together, the results of this study illustrate the correlation between metabolites and genes and provide insights into the formation of the peach-like aroma of strawberry fruits.

## 5. Conclusions

To identify the key aroma components, candidate genes, and pathways that contribute to the characteristic aroma of *F. nilgerrensis* fruits, we analyzed the fruits of *F. nilgerrensis* (with a peach-like aroma) as well as the fruits of its interspecific hybrids PA (with a peach-like aroma) and NA (without a peach-like aroma; control) at the metabolome and transcriptome levels. A total of 150 DAMs were detected. More specifically, the fruits of HA and PA contained significantly more esters/lactones (e.g., acetic acid, octyl ester, δ-octalactone, and δ-decalactone) than the fruits of NA. Hence, these metabolites may be crucial for the formation of the peach-like aroma of *F. nilgerrensis* fruits. Moreover, DEGs significantly enriched in two GO terms of fatty acid metabolic process and fatty acid biosynthetic process, and three KEGG pathways of linoleic acid metabolism, α-linolenic acid metabolism, and fatty acid degradation that may be associated with ester/lactone synthesis. Furthermore, 27 DEGs were predicted to be associated with esters and lactones biosynthesis, including *AAT*, *LOX*, *AOS*, *FAD*, *ATM1*, *EH*, *FAH*, *ADH*, and cytochrome P450 subfamilies genes. Additionally, the 35 TFs predicted to be involved in the production of aromatic compounds included bHLH, MYB, bZIP, NAC, AP2, GATA, and TCP TFs. On the basis of a co-expression analysis, differentially expressed *FAD*, *AOS*, and cytochrome P450 family genes as well as TF genes in the *NAC*, *MYB*, and *AP2* families were revealed to be closely related to δ-octalactone and δ-decalactone. The contributions of these key metabolites, pathways, and genes to the formation of the peach-like aroma of *F. nilgerrensis* will need to be experimentally verified. Nevertheless, the results of this study provide insights into the peach aroma formation of *F. nilgerrensis* fruit and may be relevant for optimizing the application of wild strawberry germplasm resources in breeding programs.

## Figures and Tables

**Table 1:** Transcriptome sequencing data for the fruit samples from *F. nilgerrensis* Schlecht. (HA) and its interspecific hybrids PA and NA.

Sample	Raw Reads (M)	Clean Reads (M)	Clean Base (G)	Q20 (%)	Q30 (%)	GC (%)
HA-1	47.30	44.37	6.66	98.17	94.54	45.75
HA-2	45.76	42.85	6.43	97.98	94.27	46.44
HA-3	47.59	44.11	6.62	97.49	92.99	46.14
NA-1	43.43	42.00	6.3	98.06	94.32	46.30
NA-2	48.95	47.23	7.09	97.95	94.14	46.54
NA-3	48.01	46.93	7.04	98.00	94.13	46.35
PA-1	45.89	43.92	6.59	98.09	94.39	46.08
PA-2	46.13	43.45	6.52	98.07	94.34	45.45
PA-3	47.79	45.60	6.84	98.05	94.27	45.97
Sum	420.86	400.45	60.09			

## Data Availability

The datasets supporting the conclusions of this article are included within the article and its additional files. The datasets used and analyzed during the current study are available from the authors on reasonable request (Aihua Wang, 118wah@163.com).

## Supplementary Materials

The following supporting information can be downloaded at: https://www.mdpi.com/article/10.3390/genes13071285/s1, Figure S1: Validation of 8 selected DEGs via qRT-PCR; Table S1: Primers used for qRT-PCR analysis.; Table S2: All 150 DAMs identified in the NA vs. HA and NA vs. PA comparisons; Table S3: Twenty-nine esters/lactones that were significantly more abundant in PA and HA than in NA; Table S4: Twenty-seven DEGs predicted to be associated with ester and lactone biosynthesis; Table S5: Thirty-five TFs predicted to be associated with aroma formation; Table S6: Analysis of the correlation between 29 esters/lactones and 27 structural DEGs; Table S7: Analysis of the correlation between 29 esters/lactones and 35 TFs.

## Abbreviations

*AAT*	Alcohol acyltransferase
*LOX*	Lipoxygenase
*AOS*	Allene oxide synthase
*FAD*	Fatty acid desaturase
*EH*	Epoxide hydrolase
*AIM1*	Hydroxyacyl-CoA dehydrogenase
*FAH*	Fatty acid hydroxylase
*ADH*	Alcohol dehydrogenase
DEG	Differentially expressed gene
DAM	Differentially accumulated metabolite
