# Supplementary material for: Transcriptomic and Metabolomic Analyses Provide Insights into the Formation of the Peach-like Aroma of Fragaria nilgerrensis Schlecht. Fruits
Source: Genes (Basel). 2022 Jul 20;13(7):1285. doi: 10.3390/genes13071285 (PMC9318527; doi:10.3390/genes13071285)
Supplement: Supplementary file 1 [file genes-13-01285-s001.zip › Table S1.pdf]

**Table S1 Primers used for qRT-PCR analysis**.

| Gene | Forward primer (5′→ 3′) | Reverse primer (5′→ 3′) | Annealing temperature (℃) |
|---|---|---|---|
| *FaActin* | CGAGCTGTTTTCCCTAGCAT | TCATCTTCTCACGATTAGCCTT | 55.0 |
| *gene-LOC101312801* | ATCCTCTGATTCACAGTTTCC | ACACTCCCAGCCGCTCAGCCT | 51.6 |
| *gene-LOC101293406* | GTGAGCCAGTAGTAATAGCAA | ATTGATAAGGATTTGCCTGG | 51.3 |
| *gene-LOC101309231* | ACGAGTGTGGCCACCACGCCT | TCCGATGTTGGAGTGGTGGCG | 63.1 |
| *gene-LOC101311794* | AGATAGAGTTATGGGGCTTGT | TCTTCCCGGTTCCTGGAATTG | 57.3 |
| *gene-LOC101309291* | AGCAGTGGAAAACCCGTATAC | TGCACATCCTAAAATGGCAG | 54.7 |
| *gene-LOC105349995* | ACCATTTCATATCATTAACAT | CCATATTGTGCCACCTATCGG | 55.8 |
| *gene-LOC105352442* | CAGTCAGCTGTTGATCATCAC | TGTGATGTCATTGACATTATT | 53.5 |
| *gene-LOC101313165* | AAGGAAGAGAATGCTGTCGA | GTGGTTCTCCTTCCTCAGCTG | 58.1 |
