# Supplementary figures and images for: Transcriptomic and Metabolomic Analyses Provide Insights into the Formation of the Peach-like Aroma of Fragaria nilgerrensis Schlecht. Fruits
Source: Genes (Basel). 2022 Jul 20;13(7):1285. doi: 10.3390/genes13071285 (PMC9318527; doi:10.3390/genes13071285)

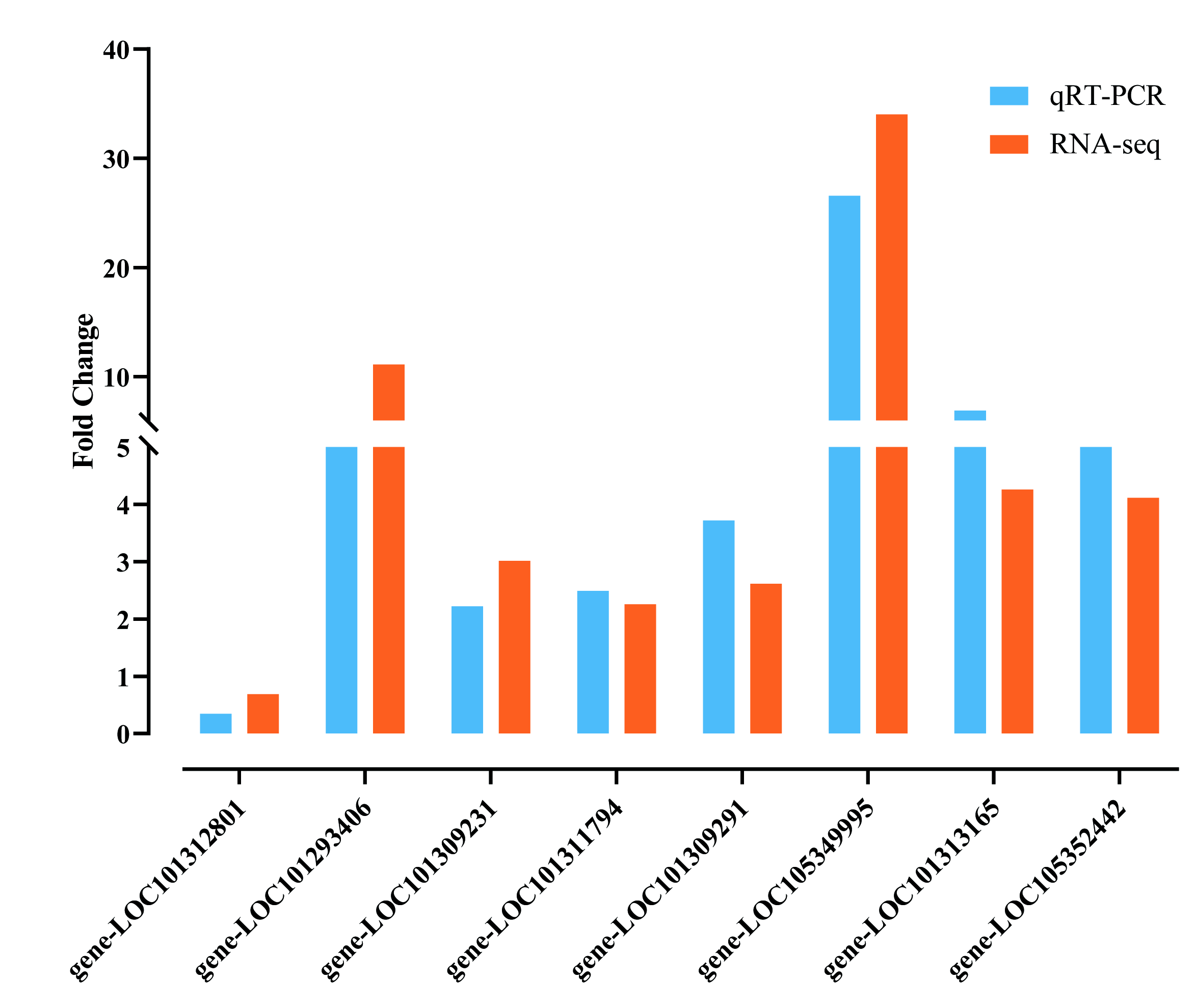

Supplement: Supplementary file 1 [file genes-13-01285-s001.zip › FigureS1.tif]
